# Public perceptions of non-pharmaceutical interventions for reducing transmission of respiratory infection: systematic review and synthesis of qualitative studies

**DOI:** 10.1186/1471-2458-14-589

**Published:** 2014-06-11

**Authors:** Emma Teasdale, Miriam Santer, Adam W A Geraghty, Paul Little, Lucy Yardley

**Affiliations:** 1 Primary Care and Population Sciences, Faculty of Medicine, University of Southampton, Southampton, UK; 2 Academic Unit of Psychology, Faculty of Social and Human Sciences, University of Southampton, Southampton, UK

**Keywords:** Qualitative, Thematic synthesis, Systematic review, Respiratory infection, Non-pharmaceutical intervention

## Abstract

**Background:**

Non-pharmaceutical public health interventions may provide simple, low-cost, effective ways of minimising the transmission and impact of acute respiratory infections in pandemic and non-pandemic contexts. Understanding what influences the uptake of non-pharmaceutical interventions such as hand and respiratory hygiene, mask wearing and social distancing could help to inform the development of effective public health advice messages. The aim of this synthesis was to explore public perceptions of non-pharmaceutical interventions that aim to reduce the transmission of acute respiratory infections.

**Methods:**

Five online databases (MEDLINE, PsycINFO, CINAHL, EMBASE and Web of Science) were systematically searched. Reference lists of articles were also examined. We selected papers that used a qualitative research design to explore perceptions and beliefs about non-pharmaceutical interventions to reduce transmission of acute respiratory infections. We excluded papers that only explored how health professionals or children viewed non-pharmaceutical respiratory infection control. Three authors performed data extraction and assessment of study quality. Thematic analysis and components of meta-ethnography were adopted to synthesise findings.

**Results:**

Seventeen articles from 16 studies in 9 countries were identified and reviewed. Seven key themes were identified: perceived benefits of non-pharmaceutical interventions, perceived disadvantages of non-pharmaceutical interventions, personal and cultural beliefs about infection transmission, diagnostic uncertainty in emerging respiratory infections, perceived vulnerability to infection, anxiety about emerging respiratory infections and communications about emerging respiratory infections. The synthesis showed that some aspects of non-pharmaceutical respiratory infection control (particularly hand and respiratory hygiene) were viewed as familiar and socially responsible actions to take. There was ambivalence about adopting isolation and personal distancing behaviours in some contexts due to their perceived adverse impact and potential to attract social stigma. Common perceived barriers included beliefs about infection transmission, personal vulnerability to respiratory infection and concerns about self-diagnosis in emerging respiratory infections.

**Conclusions:**

People actively evaluate non-pharmaceutical interventions in terms of their perceived necessity, efficacy, acceptability, and feasibility. To enhance uptake, it will be necessary to address key barriers, such as beliefs about infection transmission, rejection of personal risk of infection and concern about the potential costs and stigma associated with some interventions.

## Background

Acute respiratory infections represent a significant public health issue. They place a continual and considerable burden on public health (serious illness, reduced quality of life), overstretched healthcare services, and on public prosperity (increased absenteeism and reduced workforce productivity) [1-3]. Reducing the transmission of acute respiratory infections could therefore be extremely valuable to the general public, healthcare services and society as a whole.

Respiratory infection control comprises pharmaceutical (e.g. vaccination) and non-pharmaceutical public health interventions. Research suggests that non-pharmaceutical respiratory infection control may provide simple, low-cost, effective ways of reducing the transmission and minimising impact of acute respiratory infections in pandemic and non-pandemic contexts [4-6]. In the early stages of an emerging respiratory infection outbreak or pandemic, it is unlikely that there will be immediate and sufficient availability of a vaccine on a global scale due to the novelty of the virus [7]. Non-pharmaceutical interventions may be particularly important in the early phase of influenza pandemics, in which slowing the spread of infection could help to reduce the number of people who become infected whilst a vaccine is developed [8-10]. Minimising the spread of infection would enable the continued functioning of vital public services and lessen the socioeconomic impact of a pandemic [11].

Non-pharmaceutical respiratory infection control comprises public health interventions that communities and individuals can adopt both when well (to reduce exposure to the virus and avoid becoming infected) and when infected (to avoid affecting others and to recover from illness). Non-pharmaceutical interventions involve behaviours such as isolation e.g. staying home if feeling ill, personal protective measures e.g. covering coughs and sneezes and washing hands often with soap or hand gel, social distancing e.g. postponing or cancelling large public gatherings and using remote healthcare services (Table 1). Effective management of acute respiratory infections could involve isolation and treatment where appropriate, and advising the general public on both the pharmaceutical and non-pharmaceutical protective actions that they can take to help control the spread of infection in both pandemic and non-pandemic contexts [12].

**Table 1:** Non-pharmaceutical interventions for respiratory infection control

**Behaviour**	**Definition**
**Isolation**	Staying home if symptomatic for at least 7 days (minimising contact with other household members) to reduce peak incidence of respiratory infection.
**Personal Protective Measures (PPM)**	Hygiene and distancing behaviours to reduce an individual’s chance of catching and passing on respiratory infections
***-Respiratory hygiene***	Covering/catching coughs and sneezes using disposable tissues
***-Hand hygiene***	Washing hands regularly and thoroughly with soap and water or hand gel
***-Mask wearing***	Wearing a surgical face mask
***-Personal distancing***	Keeping a distance of about 1 metre (3 feet) from people who appear symptomatic
**Social Distancing**	Actions taken by communities to reduce social contact and to literally increase the space between people
***-in children***	Temporarily closing schools and childcare facilities
***-in adults***	Postponing or cancelling large public gatherings, altering workplace environments, e.g. offering telework or remote-meeting options.
**Remote healthcare**	Accessing website or phone line advice and support, and setting up ’Flu friends’ (if ill) rather than going to local healthcare facilities to reduce spread of respiratory infection and avoid overstretching healthcare services.

Theoretical models such as Theory of Planned Behaviour [13] and Protection Motivation Theory [14,15] emphasise the importance of perceptions and beliefs in predicting protective behaviour. Previous research has shown that how people respond to the threat of a new respiratory infection can be positively influenced by briefly addressing their beliefs about the efficacy and perceived costs of protective behaviours [16]. Improving our understanding of perceptions, beliefs and other factors likely to motivate people to adopt non-pharmaceutical interventions in both pandemic and non-pandemic contexts would facilitate the development of public health advice messages that effectively promote non-pharmaceutical respiratory infection control.

Factors that influence the adoption of non-pharmaceutical respiratory infection control include perception of risk, anxiety and efficacy beliefs (i.e. beliefs about one's ability to successfully adopt behaviours and the effectiveness of adopting behaviours in eliminating the health threat). This has been demonstrated in both anticipated and actual respiratory infection outbreaks e.g. SARS coronavirus outbreak and H1N1 2009 pandemic [17-25]. Much of the existing research on non-pharmaceutical interventions has adopted a quantitative design, which provides important information about the frequency of people's views and the potential determinants of behaviour but does not enable us to understand more about why those views are held.

Qualitative research enables an in-depth exploration of people’s beliefs and perceptions in varying contexts, and consideration of other (non-cognitive) determinants such as emotional and sociocultural factors. For example, qualitative research on how the public make sense of emerging infectious diseases suggests that, rather than rationally assessing the likelihood and severity of infection, people interpret its potential impact by drawing on similar past events to make the unfamiliar seem more familiar and by transforming abstract concepts into concrete symbols. One such mechanism is to blame ‘the other’ or other groups of people and to underestimate the risks faced by ‘the self’ [26-28]. Synthesising qualitative research aims to identify and draw together the findings of individual qualitative studies in order to generate new insights and to further interpret and understand the findings of the pool of research [29]. The process of synthesising qualitative research can provide valuable insights into the needs, preferences and experiences of the public regarding healthcare advice or interventions and as such has a significant role to play in informing the development of effective healthcare [30]. Greater understanding of how people in different circumstances make sense of public health advice on non-pharmaceutical interventions may allow us to examine where current public health communication efforts could be undermined.

To our knowledge, there has been no previous attempt to synthesise qualitative studies on non-pharmaceutical respiratory infection control. Our aim was to synthesis the qualitative literature on public perceptions of non-pharmaceutical public health interventions that aim to reduce the transmission of acute respiratory infections.

## Methods

### Search strategy and inclusion criteria

We sought papers whose primary focus was public perceptions (general public or patient groups) of non-pharmaceutical measures to reduce transmission of acute respiratory infections and/or interventions that aimed to promote non-pharmaceutical respiratory infection control. To be eligible for the review, studies had to use qualitative methods of data collection and data analysis. Studies that used a mixed methodology were included if they comprised a substantive qualitative component (i.e. the depth and breadth of the qualitative data was sufficient to form a stand-alone qualitative paper). Studies whose primary focus was the views of healthcare professionals, views of children or views about respiratory infections with no data on reduction of transmission were excluded (Table 2).

**Table 2:** Inclusion and exclusion criteria

	**Inclusion criteria**	**Exclusion criteria**
**Population of interest**	Adults ≥17years old	Health professionals, Children
**Exposure of interest**	Non-pharmaceutical respiratory infection control:	Pharmaceutical respiratory infection control:
	●Hand hygiene	●Vaccination
	●Respiratory hygiene	●Antivirals
	●Mask wearing	
	●Isolation	
	●Social distancing	
	●Remote health care	
	●Precautionary avoidance	
**Outcome of interest**	Public perspectives of respiratory infection control (including beliefs, views, concerns, understandings and emotional and sociocultural factors)	
**Study design**	Qualitative *(ethnography, grounded theory, phenomenology, focus groups, Interviews and participant observation)* and mixed methods	Quantitative

Five electronic databases were searched: Medline (1946 to January Week 3 2013), PsycINFO (1887 to February week 1 2013), Embase (1980 to 2013 Week 05) CINAHL (1982 to February week 3 2013), and Web of Science (1981 to February week 4 2013). The last search was conducted on 26th February 2013. We conducted scoping exercises to develop comprehensive search strategies for each database (Additional file 1). We adopted a pre-planned, comprehensive search strategy, rather than using iterative theoretical sampling seeking conceptual saturation, to enhance the transparency and reproducibility of the review [30]. As many electronic databases do not yet have gold standard indexing of qualitative research our search terms for qualitative research were adopted from lists of qualitative search terms from key literature [31,32]. No language restrictions or year limits were applied to the searches. Potentially relevant studies not published in English were translated by a native speaker. Although the focus of this review was non-pharmaceutical respiratory infection control, the search term ‘vaccination’ was initially included to allow for papers that included both pharmaceutical and non-pharmaceutical measures to be identified. Studies whose primary focus was vaccination alone were then excluded.

One author (ET) screened titles and abstracts of all identified papers against the inclusion criteria. Those that did not meet the inclusion criteria or were duplicates were excluded. A second author (MS) screened 10% of the title and abstracts to check that potentially relevant studies had not been missed. Full papers for the remaining identified records were retrieved and assessed for eligibility. Multiple papers from a single study were included if each paper presented unique data. Reference lists of all potentially relevant papers were reviewed and corresponding authors of included papers contacted to allow additional relevant studies to be identified.

### Quality appraisal and data extraction

Prior to synthesis, 3 authors (ET, MS & AG) extracted data and appraised the quality of included papers. Details about study design, participants (number and characteristics), type of non-pharmaceutical intervention and study context (timing, location, type of respiratory infection) were extracted into a Microsoft Word template. Papers were appraised using the Critical Appraisal Skills Programme (CASP) quality assessment tool for qualitative studies [33]. This tool enabled independent critical review of the quality of our identified studies. We employed this tool in order to systematically examine and document the strengths and weaknesses of the studies but not necessarily to exclude them. We acknowledge the potential risk that valuable new insights, grounded in data, might be missed when studies are excluded from a synthesis due to methodological flaws or lack of reporting. However, through discussion and consensus, one paper was excluded following appraisal as it reported no details about data collection, management and analysis. As such we could not be confident that this paper met our inclusion criteria of using qualitative methods of data collection and data analysis.

### Synthesis

We used thematic synthesis [34] and meta-ethnographic [35] methods to synthesise findings. Meta-ethnography uses the notion of first, second and third order constructs to synthesise qualitative papers, where first order constructs reflect data on participant views, second-order constructs are the original researchers’ interpretations of themes arising from data, and third-order constructs are the new, common themes or interpretations derived by the synthesis of second-order constructs from multiple papers. Many of the papers included in this synthesis were applied studies that offered more descriptive analyses rather than conceptually rich analyses so a meta-ethnography was not possible and thematic synthesis was employed to identify and organise the synthesis data into themes. Components were adopted from meta-ethnography (reciprocal translational analysis and refutational synthesis) in order to facilitate synthesis across heterogeneous circumstances (different respiratory infections, different non-pharmaceutical interventions and groups with very different demographic, sociocultural and illness-related characteristics). Each paper was read repeatedly to become familiar with the data. The findings from each paper (participants’ views and author interpretation of findings) were then extracted verbatim and imported into Nvivo version 9.2. Data for each paper were coded line by line by ET according to meaning and content. Coding was inductive in nature (i.e. codes were grounded in the data, reflecting the language present in original studies). The list of codes generated for each paper was then systematically compared and developed as subsequent papers were coded (i.e. codes translated into one another or new codes added to the list). A coding manual was produced by ET to ensure transparent coding of the data. Initial themes and sub-themes were developed and refined through discussion with MS, AG and LY. ET then carried out consistency checks on the text coded to each theme and sub theme and organised themes into grids and tables to be compared and juxtaposed to examine variability between studies and by infection context, populations and intervention type. ET, MS, AG and LY then reviewed and discussed the evolving coding manual further and more abstract themes were developed based on the authors’ inferences and interpretation of the data. Diagramming was then employed to explore links and illustrate the key themes within the data.

## Results

**Figure 1:**
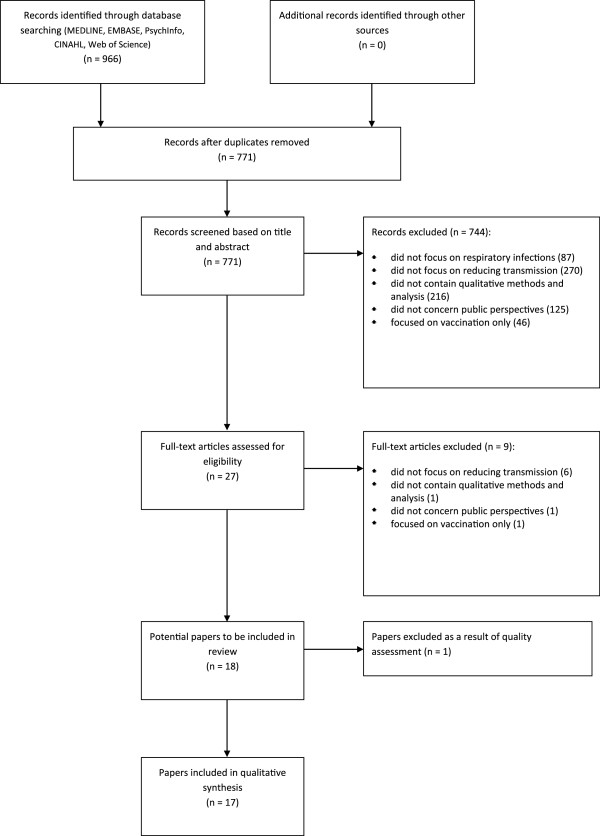
Flowchart of systematic search.

The database searches yielded 966 records in total. After screening for duplicates and eligibility, 17 papers (from 16 studies) satisfied the selection criteria and were included in the synthesis (Figure 1).

### Study characteristics

The characteristics of the 16 included studies are shown in Table 3. Studies were published between 2005 and 2012 and represented the views of 1,022 participants from 9 different countries. The majority of studies (n = 12) recruited members of the general public with varying demographics and in different contexts. Two studies focused on specific ethnic groups and two on people with a chronic illness. In terms of infection context, 10 studies focused on an actual respiratory infection outbreak/pandemic, namely SARS and the H1N1 2009 pandemic. Typically, the included studies explored multiple non-pharmaceutical interventions or combined mitigation strategies involving pharmaceutical and non-pharmaceutical interventions. Personal protective measures were most commonly studied (n = 10).

**Table 3:** Characteristics of included studies

**Study (country)**	**Infection context (timing)**	**Participants (sampling)**	**Data collection and analysis**	**Behaviour type**	**Aims**
**Cava et al. (2005)**[36,37] Canada	**SARS** (During SARS, 2003-2004)	21 adults quarantined during SARS outbreak in Toronto. (Stratified random)	Semi-structured interviews (21) Not stated (Miles & Huberman 1994) ^†^	**ISOLATION** (Quarantine)	*To explore the experience of being on quarantine for severe acute respiratory syndrome (SARS) with a focus on the relationship between perceived risk of contracting SARS and reported compliance with the quarantine order and protocols.*
**Janssen et al. (2006)**[38]USA	**Avian Flu** (Non-pandemic, 2005)	136 members of the general public. (Purposive)	Focus groups Not stated	PPM* (hygiene & vaccination)	*To test pandemic influenza messages with the public for understandability, believability/credibility, level of interest in the subject, perceived importance of the information, likelihood of action after being exposed to the information, and unanticipated consequences of the information.*
**Elledge et al. (2008)**[39] USA	**Avian Flu** (Non-pandemic, 2006)	60 members of the general public.(Not stated)	Focus groups (12) Not stated	**PPM** (hygiene)	*To determine the level of awareness of avian and pandemic flu for the county health department to develop effective communication messages*
**Jiang et al. (2009)**[40]UK & Netherlands	**SARS** (Post SARS, 2005-2006)	164 European Chinese adults living in the UK & Netherlands. (Purposive)	Focus groups (23) Framework analysis (Ritchie J, Lewis J 2003)	**PPM** (Mask wearing and personal distancing)	*To examine SARS-related risk perceptions and their impacts on precautionary actions and adverse consequences from the perspective of vulnerable communities living in unaffected regions.*
**Morrison & Yardley (2009)**[41]UK	**Pandemic Flu** (Non-pandemic, 2008)	31 members of the general public. (Purposive)	Focus groups (8) & semi structured interview (1) Thematic analysis (Joffe H, Yardley L, 2004)	**PPM** (Hygiene & personal distancing)	*To examine perceptions of infection control measures in the context of pandemic influenza.*
**Baum et al. (2009)**[42]USA	**Pandemic Flu** (Non-pandemic, 2008)	37 members of the general public. (Purposive)	Focus groups (4) Thematic analysis (Creswell 2006; Krueger 1998; Weber 1990).	**DISTANCING** (closure of schools, workplaces, public gatherings and quarantine)	*To characterize public perceptions about social distancing measures likely to be implemented during a pandemic.*
**Caress et al. (2010)**[43]UK	**H1N1 2009** (Pandemic, 2009-2010)	50 adults with a clinician-diagnosed chest problem & their family members(Purposive)	One to one interviews (20) & focus groups (3) Framework analysis (Ritchie & Spencer, 1994)	**ISOLATION & REMOTE CARE** (Social isolation, help seeking and vaccination)	*To explore and compare information needs, worries and concerns, and health-related behaviours regarding swine flu in people with respiratory conditions and their family members.*
**Yardley et al. (2010)**[44]UK	**Seasonal Flu and H1N1 2009** (Pandemic, 2009)	28 members of the general public.(Purposive)	Semi structured -think aloud interviews Thematic analysis (Braun & Clarke, 2006; Joffe & Yardley, 2004)	**PPM** (Hand washing)	*To explore attitudes towards preventive behaviours to reduce the risk of transmission of seasonal and pandemic flu in the UK in order to inform development of an intervention.*
**Sui (2010)**[45] Hong Kong	**H1N1 2009** (Pandemic, 2009)	30 chronic renal disease patients (Purposive)	Participant observation, semi-structured interviewsThematic content analysis (Liamputtong & Ezzy, 2005)	**PPM** (Mask wearing and personal distancing)	*To demonstrate the knowledge perceptions of and the preventive health behaviours toward the influenza A H1N1 pandemic among the chronic renal disease patients in Hong Kong.*
**Hilton & Smith (2010)**[46] UK	**H1N1 2009** (Pandemic, 2009-2010)	73 members of the general public. (Purposive)	Focus groups (14) Not stated (Pope & Mays 2000)^†^	**PPM** (Hygiene & vaccination)	*To examine public understandings of the swine flu pandemic, exploring how people deciphered the threat and perceived they could control the risks.*
**Ferng et al. (2011)**[47] USA	**Influenza-like illness** (Non-pandemic, 2008)	15 Hispanic females living in USA (Purposive)	Participant observation and one focus group Not stated	**PPM** (Mask wearing)	*To identify barriers to mask wearing for influenza-like illness and to examine the factors associated with the willingness to wear masks among households.*
**Nizame et al. (2011)**[48] Bangladesh	**Respiratory infections** (Non-pandemic, 2008-2009)	178 members of the general public. (Purposive)	Interviews (34) & Focus Groups (16) Thematic content analysis	**PPM** (Hand and respiratory hygiene	*To explore community perceptions on respiratory infections, why they occur, how they are spread, and the preventive measures that people take to protect themselves and their families.*
**Teasdale & Yardley (2011)**[49] UK	**H1N1 2009** (Pandemic, 2009)	48 members of the general public. sive)	Focus groups (11) Thematic analysis (Braun & Clarke, 2006; Joffe & Yardley, 2004)	**ISOLATION & REMOTE CARE** (Social isolation, remote health care & vaccination)	*To explore people’s beliefs, perceptions, reasoning, and emotional and contextual factors that may influence responses to government recommendations for managing flu pandemics.*
**Gray et al. (2012)**[50] New Zealand	**H1N1 2009** (Pandemic, 2010)	80 members of the general public. (Purposive)	Focus groups (8) Thematic analysis (Braun and Clarke, 2006)	**PPM & DISTANCING** (social isolation, social distancing & vaccination)	*To provide qualitative data about community responses to key health messages in the 2009 and 2010 H1N1 campaigns, the impact of messages on behavioural change and the differential impact on vulnerable groups in New Zealand.*
**Rodriguez (2012)**[51] Spain	**H1N1 2009** (Pandemic, 2010)	51 members of the general public.(Purposive)	Focus groups (10) Not stated	**PPM** (Hygiene & vaccination)	*To explore the views of the general population, the risk groups and medical personnel on the H1N1 influenza epidemic of winter 2009-2010.*
**Seale et al. (2012)**[52] Australia	**Seasonal Flu and H1N1 2009** (Pandemic, 2010)	20 university students in New South Wales. (Convenience)	Semi-structured interviews Not stated	**PPM & DISTANCING**(Hygiene, social distancing and isolation)	*To examine the knowledge, attitudes, risk perceptions, practices and barriers towards influenza and infection control strategies.*

*PPM – Personal Protective Measures † Analytical method was not explicitly stated, however relevant reference was provided.

### Key themes

**Figure 2:**
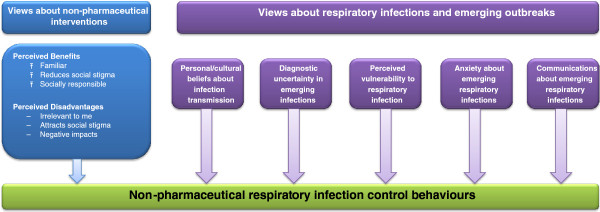
Themes and sub-themes of synthesis data.

The synthesis findings comprised three parts: 1) the ways in which the public evaluate non-pharmaceutical interventions, 2) public beliefs about respiratory infections and emerging outbreaks, 3) presentation of advice on adopting non-pharmaceutical respiratory infection control i.e. public preferences for how and by whom non-pharmaceutical respiratory infection control advice is presented. This paper focuses on the first two parts as they were relevant to the original aim of the review of understanding public perceptions of non-pharmaceutical interventions rather than views about how advice about adopting these behaviours is presented. Seven key themes were identified: *perceived benefits of non-pharmaceutical interventions, perceived disadvantages of non-pharmaceutical interventions, personal and cultural beliefs about infection transmission, diagnostic uncertainty in emerging respiratory infections, perceived vulnerability to respiratory infection, anxiety about emerging respiratory infections and communications about emerging respiratory infections* (Figure 2). Table 4 presents the definitions of each theme and an index of which themes were present in each paper. The themes and corresponding quotes are described below. Quotes are labelled with study reference, population, infection context and location.

**Table 4:** Contribution of key themes from each study

	**Theme Sub-theme**	**Summary definition**	**Study reference by infection context and study population**						
			**SARS**		**Non-pandemic**		**H1N1 2009 pandemic**		
			**S1**	**S2**	**N1**	**N2**	**P1**	**P2**	**P3**
1	Perceived benefits of non-pharmaceutical interventions								
a	Hand and respiratory hygiene is common sense/familiar	Hygiene behaviours are seen as familiar and acceptable in varying contexts and populations			[38,39,41,48]		[44,46,50-52]	[50]	[43]
b	Mask wearing demonstrates responsibility and reduces stigma	Mask wearing is seen as a way of visibly demonstrating one’s desire to protect self and others from infection, which can in turn reduce social stigma experienced.							[45]
c	Social isolation and distancing are socially responsible actions	Isolation and distancing are believed to be socially responsible actions and seen as necessary for the protection of society as a whole	[36]		[41,42]				
2	Perceived disadvantages of non-pharmaceutical interventions								
a	Hand washing for respiratory infection control is irrelevant	Additional hand washing behaviours are seen as irrelevant by those who class themselves as regular hand washers			[41]		[44,46]		
b	Hand washing and mask wearing can attract social stigma	Hand washing and mask wearing are perceived as socially unacceptable due to the potential to attract discrimination and embarrassment		[40]	[41]	[47]	[44,52]		
c	Non-pharmaceutical behaviours have negative personal and socioeconomic impacts	Perceived physical, practical, emotional and socioeconomic costs of isolation social distancing, mask wearing and hygiene behaviours	[36,37]		[41,42,48]	[47]	[44,49-52]	[50]	
3	Personal/cultural beliefs about infection transmission								
		Common beliefs about respiratory infections are caught and spread e.g. via air, from symptomatic others and in cold temperatures	[36]	[40]	[41,48]		[44,46,49,52]		[43]
4	Diagnostic uncertainty in emerging respiratory infections								
		Identifying symptoms of and having to diagnose infection in an emerging respiratory infection is seen as confusing and concerning and can lead to uncertainty about when to adopt infection control	[36,37]				[46,49-52]		[43]
5	Perceived vulnerability to respiratory infections								
a	Perceived health status	Evaluating one’s vulnerability to respiratory infection in terms of own perceived health status and the health of others					[44,46,50-52]		[43,45]
b	Proximity to the origin of outbreak	Evaluating susceptibility to a new respiratory infection in terms of geographical proximity to the origin of the outbreak and type of living environment		[40]	[38,39,41,42]		[46,50,52]		
6	Anxiety about emerging respiratory infections								
a	Decreasing anxiety over the course of an outbreak	Initial anxiety in an outbreak decreases over the course of the outbreak as public reassess the risk/impact of a new respiratory infection according to personal experience vs. information presented in the media		[40]			[46,49,51]		[43]
b	High anxiety if perceived to be more vulnerable	Greater anxiety experienced during H1N1 by those who perceived themselves to be more vulnerable to infection.					[46,51]		[43]
c	Low anxiety	Low levels of worry experienced during an emerging respiratory infection outbreak					[46,49,50]		[43]
7	Communications about emerging respiratory infections								
a	Media reporting of information on new respiratory infection outbreaks is seen overhyped	People appraise the credibility of information/communications about a new respiratory outbreak in terms of consistency of information and perceived exaggeration compared to actual/previous experience	[36,37]	[40]	[39,41]		[46,49-52]		[43]
b	Official communication about new respiratory infection outbreaks is not reliable (threat is downplayed)	Some people’s evaluation of information influenced by scepticism about level of detail presented (i.e. not being given all the facts)			[39,42]		[50]		[43]

S1: SARS general public, S2: SARS ethnic groups; N1: Non-pandemic general public, N2: Non-pandemic ethnic groups; P1: H1N1 general public, P2: H1N1 ethnic groups, P3: H1N1 patient groups.

### *Perceived benefits of non-pharmaceutical interventions*

Some personal protective measures, such as hand-washing and respiratory hygiene, appeared to be widely acceptable methods for preventing respiratory infection transmission across several studies. Studies conducted prior to and during the H1N1 2009 pandemic indicated that people were generally very familiar with hand and respiratory hygiene behaviours (e.g. hand washing, cough/sneeze etiquette) and viewed them as usual, acceptable, common-sense actions to reduce infection transmission.

*“Mainly they tell you to wash your hands....Cover your mouth when you cough.......And don’t share hankies, they say, yeah.” (Pacific Peoples, H1N1 2009, New Zealand)*[50]

*“I already do the necessary, like washing my hands after the loo, which is, well, common practice and basic hygiene and being aware of it” (General public, non-pandemic, UK)*[44]

For people who perceive themselves to be at high risk of catching and spreading respiratory infections, mask wearing was seen not only as an effective but also as a visibly demonstrative method of respiratory infection control, which could reduce social stigma. Chronic kidney disease (CKD) patients in Hong Kong saw mask wearing as a useful way to demonstrate their desire to be socially responsible and protect themselves and others during H1N1 2009 pandemic. This was related to social stigma experienced by CKD patients in Hong Kong during SARS, where they were perceived publically as high risk ‘super spreaders’ of infection.

*“I wore facemasks all the time to tell others that I was responsible about my own and about others’ health. This strategy really worked as my friends and colleagues did not isolate me too much since then. I think I can use the same strategy in this swine flu to make others feel more comfortable with me, because I can show that even if I carry a lot of flu virus, I have already tried my best to protect their health by wearing facemasks.” (CKD patient, H1N1 2009, Hong Kong)*[45]

Similarly, isolation and social distancing behaviours were viewed as acceptable in some contexts as a way of being socially responsible (i.e. protecting wider society). For example, being isolated or quarantined during SARS was accepted as necessary in order to protect others from infection. Studies conducted in a non-pandemic context indicated that the general public viewed home isolation, closure of schools and public gathering places (e.g. religious centres), and travel restrictions during an emerging respiratory infection outbreak as important for the protection of society as a whole, and would adopt these behaviours in principle for the greater good.

*“We’re all trying to be good citizens. And we’re all trying to help, you know, other people by making sacrifices like being in quarantine.” (General public, SARS 2005, Canada)*[36]

*“. . . you know, if you can’t go to church for one week because everyone’s sick, then you know, call everybody on the phone or something. Do something different for the good of everybody else; you may have to suffer a little bit here and there.” (General public, non-pandemic 2009, USA)*[42]*.*

### *Perceived disadvantages of non-pharmaceutical interventions*

Non-pharmaceutical respiratory infection control was seen as problematic for some in terms of its perceived irrelevance. In particular, studies conducted pre and during H1N1 2009 pandemic suggested that advice to carry out additional hand washing or to focus on hand washing solely or intentionally for respiratory infection control was not seen as necessary or relevant to members of the general public who perceived themselves to be ‘regular hand washers’ (i.e. already regularly wash their hands for basic hygiene). This highlights the habitual, ingrained nature of hand washing behaviour and the firmly established social norms around when is acceptable to wash hands (e.g. after going to the toilet), which are likely to influence the perceived acceptability of hygiene behaviours [53].

*“I don’t think that anybody washes their hands more than what they already do. You only wash your hands at normal intervals that I think you would normally, like if you're eating, after you've been to the toilet, etcetera.” (General public, non-pandemic, UK)*[41].

*“I can’t say I’ve changed anything cos I already do my hand washing, so others might benefit from this advice more really.” (General public, H1N1 2009 pandemic, UK)*[46].

Potential to attract social stigma and cause embarrassment or discrimination was another perceived downside of adopting personal protective measures. Concerns about frequent hand-washing attracting social stigma (being perceived as overly fastidious or obsessive) or causing offence (insisting on hygienic behaviours in others) were evident in various infection contexts. Likewise, it appeared that mask wearing, although seen by some as an effective precaution, could generate concern about attracting discrimination. This was due to the presence of a mask being seen to explicitly indicate infection/illness in the wearer.

*”If I’d said [when meeting friends for lunch] ah, before we touch any food we must all go and wash our hands, I’m not sure what everybody’s reaction would’ve been … I think people would’ve looked at me as if I’m slightly mad. ”(General public, H1N1 2009, UK)*[44].

*“Going out to the streets with a mask, people would stare at you as if you were contagious, but what they don’t know is that the mask is also protecting us from them” (Hispanic female, non-pandemic, USA)*[47].

“.. .is he ill or is he dangerous something like that?

*.. .like the old leprosy people in Europe.. .” (Domestic student, H1N1 2009, Australia)*[52].

Other perceived drawbacks revolved around the potential negative impact of adopting non-pharmaceutical respiratory infection control. Common perceived barriers to carrying out personal protective measures appeared to relate to personal impacts such as the perceived physical discomfort of mask wearing (e.g. difficulties breathing whilst wearing masks), and the perceived discomfort and impracticalities of hand and respiratory hygiene (e.g. dry, sore hands and noses from frequent hand washing and tissue use; not having tissues when you need them and inconvenience of frequent hand-washing).

*“It is hard to breathe with the mask. It is uncomfortable around chin area because I am sweating and the mask feels damp” (Hispanic female, non-pandemic, USA)*[47].

*“People don’t wash their hands after sneezing and coughing. Is it possible to wash hands frequently? If you sneeze 100 times, will you wash your hands 100 times? But we should wash our hands before taking a meal.”(General public, non-pandemic, Bangladesh)*[48].

Common perceived barriers to social isolation and personal distancing from those who were symptomatic seemed to relate to perceived emotional costs. For example, people who were quarantined during SARS reported feeling segregated and stigmatised for being infected.

*“Well I didn’t know I was going to get into this, but I actually feel like crying just to think about it, because I’m sure you saw the movie Ben Hur. I thought of that movie all the time while I was in quarantine because I remember the part of him going and looking for his sister and his mother, where they had that . . . sickness, leprosy. And they could not be with the rest of the people. They were down in a valley where all these people were and that’s how I felt. I was separate from the world.” (General public, SARS 2005, Canada)*[37].

Furthermore, personal distancing was viewed as unacceptable within households and some cultural groups as it may limit social interactions which were perceived as socially and culturally necessary. For Maori people in New Zealand during H1N1, concerns about being able to continue to observe specific cultural practices and greeting protocols seemed to outweigh the perceived need to adopt personal distancing behaviours. Similarly, studies conducted pre-H1N1 and during SARS, suggested that the perceived need or wish to care for sick (isolated) loved ones can override any concerns about self-protection and personal distancing.

*“My older sister said something about me having a really bad cold that weekend. And that’s only when I realized I could have got it. And I could have been very, very sick. I could have died. But it never, ever came to mind. We were so focused on her.” (General public, SARS 2005, Canada)*[36].

“But don't you have some kind of duty, or at least I think I do to look after that person.

*What if it’s a baby you've got to look after you can't do it can you?”(General public, non-pandemic 2009, UK)*[41].

People also seemed to consider the feasibility of distancing behaviours (social isolation and social distancing) in terms of the economic impact, both on a personal and societal level. Common perceived obstacles to staying home if sick and social distancing (such as school closures) during the H1N1 2009 pandemic were economic pressures to continue to work and concerns about familial and workplace commitments, and the wider adverse socioeconomic economic impact.

*“The girl downstairs who got swine flu, works in my office, came to work three times with swine flu because she was bored at home, felt that there were things that needed doing in the office, and felt guilty at being away for so long. She came in, not being able to find a manager, hang around for three hours and then got sent home again” (General public, H1N1 2009, UK)*[49].

*“. . if they keep your kids home from school so you can’t work, people are going to go, ’I can’t do that, you know, I have to go to work, I have to have somebody take care of my kids’ . . . some people might choose to keep their kids home from school if they had that luxury, but too many people now don’t.” (General public, non-pandemic, USA)*[42].

*“If you shut down the schools though, you’ve basically shut down the economy because you’d have to have, then people would have to stay home so you’re affecting a lot more than people getting sick, you’ve just affected a huge financial working to the bulk of the country. That’s a big decision.” (General public, non-pandemic, USA)*[42]*.*

### *Personal and cultural beliefs about infection transmission*

Common personal and cultural beliefs about how respiratory infections are caught and spread were evident across various infection contexts. Typically, respiratory viruses were seen as transmitted by air, caught via proximity to symptomatic others and more likely to be spread in cold ambient and water temperatures. Such beliefs are likely to influence the perceived efficacy of non-pharmaceutical interventions, particularly social isolation.

*“I’ve always thought that on an aeroplane, because they re-circulate the air, there’s a chance that viruses and things are perhaps recycled through the air or ventilation system.” (General public, H1N1 2009, UK)*[44]

*“Just because like you haven’t got any symptoms doesn’t mean you are not carrying the germ. You know, everyone thinks that umm you know if someone’s got a cold and you stand, don’t stand near me, but just by talking to them you’ve probably picked up the germ. So I don’t really see the point in staying at home if you’ve got the symptoms because the chances are everyone has already come into contact with it anyway” (General public, H1N1 2009, UK)*[49].

*“A person should not be in contact with cold water for a long time to avoid getting cold/cough.” (General public, non-pandemic, Bangladesh)*[48].

### *Diagnostic uncertainty in emerging respiratory infections*

People seemed to acknowledge that a degree of uncertainty is to be expected in an emerging respiratory infection such as SARS. However, during the H1N1 2009 pandemic, they expressed doubts about their ability to identify symptoms (e.g. distinguishing pandemic flu symptoms from seasonal flu symptoms) and concerns at having to self-diagnose or make their own judgement about the presence of infection. Such diagnostic uncertainty seemed to be exacerbated for people who had other health concerns. This suggests that people do not see it as their role to self-diagnose in an emerging respiratory infection outbreak, which could influence the likelihood of implementing non-pharmaceutical interventions such as social isolation and using remote healthcare when symptomatic.

*“I mean, the big thing is what are the symptoms, particularly what are the unique symptoms to whatever the pandemic is, that differentiates it from regular flu, or a cold? And how infectious is it, and what’s the mechanism of infection” (General public, H1N1 2009, New Zealand)*[50].

*“I think the vagueness of the symptoms could be confused with perhaps ordinary flu or just your condition really. You know, if you’ve got COPD, then it’s not necessarily swine flu at all. And I don’t really know how you can say it’s swine flu without having any tests [others in group agreeing].” (Chest patient, H1N1 2009, UK)*[43].

*“I think probably the most difficult part is to decide whether you have swine flu or not. So lots of people will be confused, when they have symptoms, what should they do” (General public, H1N1 2009, UK)*[49].

### *Perceived vulnerability to respiratory infection*

In general the public appeared to accept the existence of risk to the community in an emerging respiratory infection outbreak. However, some may view themselves as less vulnerable or more capable than ‘others’ who they identify as high risk. During the H1N1 2009 pandemic the public seemed to evaluate their vulnerability to respiratory infection in terms of perceived health status and their proximity to the origin of outbreak (both in terms of geographical distance and perceived differences in living environments). A common belief expressed was that ‘others’ were at increased risk of infection, including people with chronic health problems, those in particular occupations (e.g. teachers), and those with impaired immune systems. This reflects a typical way of dealing with a health risk and its negative impact by “othering” i.e. blaming or differentiating the self from ‘the other’ or other groups of people and consequently denying personal risk [26]. This may result in people viewing non-pharmaceutical interventions to minimise the spread of infection in an emerging outbreak as more applicable to others than to themselves.

*P1: “I’m personally not worried. I think my immune system is working well and I’m not in the situation of having an illness. P2: Same. I know like thousands of people have got it, but…I don’t personally feel at risk.” (General public, H1N1 2009 pandemic, UK)*[46].

Similarly, in non-pandemic contexts the public appeared to focus on their lack of geographical proximity to the perceived origins of an emerging respiratory infection outbreak. A common belief was that a novel respiratory virus was unlikely to originate in a ‘modern, developed country’ so they would have more time to prepare and cope better if and when it reached them. For some, the perceived lack of geographical proximity to the outbreak seemed to result in a perceived lack of immediacy of risk, which suggests that risk of infection would need to be locally imminent (evident) before advice messages would be considered and behaviours adopted.

P2: “You've gotta think, I think we're a quite clean country compared to other places like I said it will be a less.

P4: Developed P2: Developed country I think that develops it first P1: And that spreads it quicker P4: And that will educate us.

*P1: We can get a vaccine off of them, sounds nasty but it's true, it's how the world works” (General public, non-pandemic, UK)*[41].

*“. . . that right now it’s in some third world country and it may come here. I don’t think that’s going to be good enough. I think there’s going to have to be some indication that it is actually in your own community before you take steps as drastic as shutting down anything.” (General public, non-pandemic, USA)*[42]*.*

In a pandemic context, the concept of geographical proximity was challenged. Instead, it was recognised that respiratory viruses could spread worldwide quite rapidly and to more geographically remote locations due to air travel.

*“Well we think that we’re different because we’re far away. But actually, if you think of how people travelled here, it’s the biggest factor for it always, because everyone who comes here comes in an aeroplane, pretty much. And they come from everywhere.” (General public, H1N1 2009, New Zealand)*[50].

During the H1N1 2009 pandemic the public also appeared to evaluate their susceptibility to a new respiratory infection in terms of their own living environments compared to the living circumstances where they believed a novel respiratory infection was likely to emerge (e.g. low hygiene levels, high population density, poor border control and health systems).

*“It won’t be like ones in the past, years ago. There’s so much medical research been done. And things are cleaner, better housing, people are cleaner and the streets are cleaner so a pandemic wouldn’t be so bad now” (General public, H1N1 2009 pandemic, UK)*[46].

*“.. ..some cultures where they have let say more respect for traditional medicine than modern medicine.. .. …are also going to be a problem.. .that’s why lots of pandemic in say Asia and Africa, and not so much in Europe or America” (International postgraduate student, H1N1 2009, Australia)*[52].

This seems to reflect a belief that it ‘won’t happen to me, it happens to others, elsewhere’ and suggests that the public do not rationally evaluate their risk of infection but actively try to distance themselves from the threat by delineating themselves from others and other circumstances which are associated with risk of infection. This may lead to underestimating personal risk in an emerging respiratory infection outbreak and feeling that advice to adopt non-pharmaceutical interventions is not relevant.

### *Anxiety about emerging respiratory infections*

The novelty factor (shock of the new) appeared to affect public anxiety in the early stages of an emerging outbreak. During SARS and the H1N1 2009 pandemic public anxiety typically evolved over time from an initial state of anxiety gradually decreasing to the point of people making light of it as the public’s familiarity with the outbreak or respiratory infection increased through personal experience or knowing someone who had contracted it. This could be a further example of people distancing themselves from threat in order to cope with the novel health threat.

*“I think everyone has taken it a lot less seriously now than when it was first like, when it was first thing, then everyone was like ahhh, we have this swine flu and everybody is going to die but not now, it’s like if someone coughs, “ha swine flu!”, you know, no one cares now”(General public, H1N1 2009, UK)*[49].

*“There is so much joking about it, like I was on the train and this guy just coughed, and he was like, ’it’s ok, it’s not mutated, I don’t have swine flu’ and everyone just laughed. No, I don’t think people are worried about it” (General public, H1N1 2009 pandemic, UK)*[46].

For some, particularly those who were regarded to be more vulnerable to infection (e.g. people with a chronic illness, pregnant women, mothers of young children), the novelty of the virus and in particular their perceived lack of protection from it seemed to contribute to their worry.

*“[with seasonal flu] we all feel quite safe because we’ve got a protection and we know ordinary seasonal flu can be serious. But we’ve got our jab and it’s protected us. And suddenly there’s a flu out there what there hasn’t been a jab for, and we can catch it as quick as anybody else. And nobody quite knows really what effect it’s going to have on us and I think this has been some of it, because right at this time we’re vulnerable, we’ve no protection given us. And we all feel as we need that protection to get through this … And I think that’s making us worry.” (Chest patient, H1N1 2009, UK)*[43].

In contrast, some people with other health issues expressed less worry about becoming infected as they saw their current health issues as more pressing.

*“I can’t eat properly and while I’m eating I’m gasping for breath … so swine flu is the least of my worries, if you know my meaning … this [chest problem] is the priority. If I can get this right, if I can at least walk a little bit more than I can do now, I’d be happy.” (Chest patient, H1N1 2009, UK)*[43].

### *Communications about emerging respiratory infections*

Diminishing anxiety over the course of an outbreak seemed to be influenced by people’s views about communications about an emerging respiratory infection outbreak. Whist some people felt that they were not given all the facts during the H1N1 2009 pandemic or that its severity was prematurely downplayed, generally the public were quite sceptical about the way information on the new respiratory infection was presented to them (especially by the media). Typically, communication efforts are seen as unreliable, premature, inconsistent, sensationalist and unduly alarmist.

*P1: they’ve [the media] set about and managed to get everyone, or the majority of people into quite a panic about the whole thing. P2: They do on purpose whip up panic and anxiety in people” (General public, H1N1 2009 pandemic, UK)*[46]

*“At the beginning sensationalism -a blast of information-, followed by information in small doses and poor, bad information” (Chronically ill patient, H1N1 2009, Spain)*[51]

*“…the minister said one thing, someone else said another thing, Sarkozy said he was going to get everyone vaccinated, the German [leader] would say whatever, hence, we were getting so much contradictory information” (General public, H1N1 2009, Spain)*[51]

*“I feel they’ve a lot of hype with a lot of things, not just the swine flu, and particularly the media, they like to blow things up, don’t they? They like to scare people really. On the other hand I suppose scaring people is only one way to get them to move.” (Chest patient, H1N1 2009, UK)*[43]

A common belief was that media reporting in an emerging outbreak is over-hyped or can amplify the risks. It appeared that the public evaluated their personal risk by comparing personal experiences with the ‘official’ information they have been given. When this didn’t match up to actual experiences, the public doubted the credibility of the information being presented and further doubted the media as a reliable information source. This inconsistency may lead to public fatigue about respiratory infection communications and a blunting of advice messages on non-pharmaceutical interventions. Doubts about the perceived credibility and trustworthiness of information about a new respiratory infection outbreak are likely to influence public behavioural responses to an emerging respiratory infection outbreak and may also lead to people to disregard future advice.

*“It became reasonably clear reasonably quickly last time that hundreds and thousands and millions weren’t dying. Even when they kept on sort of saying things were happening, and then you saw the numbers, it just didn’t add up.” (General public, H1N1 2009, New Zealand)*[50]

*“Before that we had avian flu, and before that SARS and they were all, this is the end of the world. Don’t travel, don’t eat chickens, watch out for the dead duck in the street. And yeah, it raises your expectations and then nothing happens, and then next time it comes along you are just more cynical” (General public, H1N1 2009, UK)*[49]

## Discussion

### Main findings

Some aspects of non-pharmaceutical respiratory infection control were viewed favourably by participants in these studies due to their familiarity, potential to reduce social stigma and capacity to demonstrate social responsibility or community mindedness. Hand hygiene and respiratory hygiene, in particular, appeared to be well-established and accepted concepts in the minds of these participants. Doubts and concerns existed about the perceived relevance of non-pharmaceutical respiratory infection control, its potential to attract social stigma and its perceived adverse impact (physically, emotionally and socioeconomically). There were particular concerns about personal distancing and the wearing of masks in some contexts. The synthesis also suggested that the perceived necessity, efficacy and feasibility of adopting non-pharmaceutical respiratory infection control may be influenced by personal and cultural beliefs about respiratory infection transmission, perceptions and feelings about vulnerability to respiratory infections, and concerns around self-diagnosis of respiratory infections and the communication of reliable information about respiratory infections in emerging respiratory infection outbreaks.

### Interpretation of findings in relation to previously published work

Synthesising the qualitative literature highlighted the ways in which the public evaluate the feasibility of non-pharmaceutical respiratory infection control. Instead of passively accepting non-pharmaceutical interventions that are recommended to reduce the transmission of respiratory infections, the public actively consider the perceived costs and benefits of adopting non-pharmaceutical interventions and reflect on their beliefs about and feelings towards respiratory infections and emerging outbreaks.

Widespread public endorsement of non-pharmaceutical respiratory infection control was highlighted by the synthesis. However, such endorsement of non-pharmaceutical interventions, particularly hygiene, may not accurately and transparently reflect people’s beliefs about these behaviours. Agreeing with the socially desirable position of taking action to protect oneself and others from respiratory infection may instead reflect a more perfunctory or socio-linguistic function. Indeed, by demonstrating that you know and subscribe to what is accepted as ’common-sense’ correct behaviour could serve the purpose of affirming membership of a particular sociocultural group e.g. ‘good citizens’ [54,55]. Given the inconsistency between the socially desirable assertions about hygiene and social distancing behaviours and the numerous perceived barriers and costs of the behaviours that are subsequently raised, this seems quite probable.

One reported barrier was diagnostic uncertainty in emerging respiratory infections. In particular, public concern seems to exist about identifying symptoms and making a judgement about the presence of a respiratory infection. The public do not see it as their role to self-diagnose and feel uncomfortable about taking on the perceived role of the doctor. This finding is consistent with previous research suggesting that members of the general public do not feel comfortable with making medical decisions [56-58]. In an emerging respiratory infection outbreak, promotion of self-diagnosis and accessing remote healthcare is vital to reduce spread of respiratory infection and avoid overstretching healthcare services. Further research is required to explore how public confidence in remote healthcare could be improved.

Socio-economic barriers to adopting social distancing behaviours were also highlighted in the synthesis. This is consistent with previous research that has shown lack of access to child care and financial barriers with regards to social isolation exist, particularly for those on low incomes [59-61]. Difficulties in following non-pharmaceutical interventions faced by those with fewer resources has the potential to exacerbate the socio-economic differences in the public health impact of a respiratory infection, particularly in an emerging outbreak [62].

Another potential barrier to adopting non-pharmaceutical interventions appears to be the denial of personal risk. Although everyone is potentially at risk of respiratory infection, the public seem to deal with the threat of infection by attributing vulnerability to ‘other, less good’ groups of people. This process of ‘othering’ is consistent with previous qualitative research on how the public make sense of emerging infectious diseases [27,28]. Such denial could be seen literally as public ignorance or erroneous evaluations of risk of infection. However, denial of personal risk also means asserting that one is not in the group of people who are vulnerable or in danger due to either their risky, unhygienic behaviour or being weak (e.g. due to illness or age) - so rather than a misunderstanding of risk this can reflect a means of coping with the threat of infection. This interpretation suggests an important role of emotional and sociocultural factors in shaping individual ways to perceive and react to respiratory infection risk, rather than assuming that people’s perceptions of respiratory infection risk are purely an individual, rational, cognitive process [26,63].

### Strengths and limitations of the synthesis

Our systematic review and synthesis of qualitative studies on non-pharmaceutical respiratory infection control is novel. It provides helpful insight into the ways in which the general public process the risk of respiratory infection and evaluate the feasibility of non-pharmaceutical respiratory infection control. We used rigorous methods of systematic review and referred to the ENTREQ statement [64] to facilitate clear reporting of our synthesis of qualitative research. Although we contacted all authors to obtain any further studies, we did not comprehensively search the grey literature, which may have excluded some relevant literature. The review is clearly limited to the perceptions of participants in the included primary studies, which were typically conducted in more developed countries and predominantly explored perceptions of personal protective measures. However, the synthesis did incorporate people’s perceptions of different non-pharmaceutical respiratory infection control behaviours in varying respiratory infection contexts and can offer a higher level of conceptual thinking about public responses to non-pharmaceutical respiratory infection control across different contexts. Nevertheless, it is acknowledged that the process of synthesising qualitative studies is inherently interpretive. Our synthesis is one possible interpretation of the data. It is entirely possible that another research team may generate another interpretation of this set of studies.

### Implications for future research, policy and practice

Adoption of non-pharmaceutical public health measures is likely to be improved by addressing common public beliefs and concerns about the necessity, efficacy, acceptability and feasibility of non-pharmaceutical respiratory infection control. To maximise adoption of non-pharmaceutical interventions to reduce transmission of respiratory infections it may be necessary to find ways that allow people to associate non-pharmaceutical respiratory infection control with a positive identity rather than a negative or vulnerable identity, i.e. viewing non-pharmaceutical respiratory infection control as behaviours to be adopted by all, not just as actions for those perceived as more vulnerable to infection. For example, positive framing of advice messages around maintaining well-being rather than avoiding infection might improve the perceived relevance of non-pharmaceutical respiratory infection control to those who do not acknowledge that they could be at risk of infection. Further research is needed to clarify how best to reframe advice.

## Conclusions

People engage in an active process of evaluating non-pharmaceutical public health measures to reduce the transmission of acute respiratory infections in terms of their feasibility, credibility and costs. Some aspects of non-pharmaceutical respiratory infection control are seen as familiar and socially responsible actions to take. However, some members of the public have doubts about the relevance of non-pharmaceutical respiratory infection control, its adverse impact and potential to attract social stigma. Potential barriers include beliefs, perceptions and feelings towards respiratory infections and concerns around self-diagnosis and communications in emerging respiratory infection outbreaks. Communication efforts may be improved by addressing such barriers and concerns.

## Competing interests

The authors declare that they have no competing interests.

## Authors’ contributions

ET helped develop and refine the protocol, carried out the systematic review, data extraction, quality assessment and data analysis, and drafted the manuscript. MS and AG helped develop and refine the protocol, carried out data extraction and quality assessment, provided support with data analysis and contributed to writing the manuscript. LY helped develop and refine the protocol, provided support with data analysis and contributed to writing the manuscript. PL contributed to gaining initial funding, helped develop and refine the protocol and contributed to writing the manuscript. All authors read and approved the final manuscript.

## Pre-publication history

The pre-publication history for this paper can be accessed here:

http://www.biomedcentral.com/1471-2458/14/589/prepub

## Supplementary Material

Additional file 1 Systematic review search history: examples from MEDLINE and CINAHL lowchart of systematic search. Click here for file
