# Supplementary material for: Public perceptions of non-pharmaceutical interventions for reducing transmission of respiratory infection: systematic review and synthesis of qualitative studies
Source: BMC Public Health. 2014 Jun 11;14:589. doi: 10.1186/1471-2458-14-589 (PMC4063987; doi:10.1186/1471-2458-14-589)
Supplement: Additional file 1 — Systematic review search history: examples from MEDLINE and CINAHL lowchart of systematic search. [file 1471-2458-14-589-S1.doc]

**Additional file 1**

**Systematic review search history: examples from MEDLINE and CINAHL**

**MEDLINE**

Database: Ovid MEDLINE(R) <1946 to January Week 3 2013>, Ovid MEDLINE(R) Daily Update <January 28, 2013>, Ovid MEDLINE(R) In-Process & Other Non-Indexed Citations <January 28, 2013>, Ovid OLDMEDLINE(R) <1946 to 1965>

Search Strategy:

--------------------------------------------------------------------------------

1 *Qualitative Research/ or qualitative.mp. (111305)

2 formative research.mp. (440)

3 ethnograph*.mp. (5178)

4 Phenomenol*.mp. (13875)

5 grounded theory.mp. (4926)

6 thematic analysis.mp. (2927)

7 content analysis.mp. (9435)

8 Focus Groups/ (14243)

9 Interview, Psychological/ or Interview/ (34944)

10 Observation/ (4291)

11 purposive sample.mp. (1433)

12 narrative.mp. (9165)

13 1 or 2 or 3 or 4 or 5 or 6 or 7 or 8 or 9 or 10 or 11 or 12 (188437)

14 Infection Control/hi, is, mt, og [History, Instrumentation, Methods, Organization & Administration] (8889)

15 Health Promotion/ or protective behav*.mp. (48313)

16 preventive behav*.mp. (764)

17 avoidance behav*.mp. (2671)

18 Hand Disinfection/ (4120)

19 Hand Hygiene/ (19)

20 respiratory hygiene.mp. (33)

21 cough etiquette.mp. (19)

22 sneeze etiquette.mp. (1)

23 social distancing.mp. (147)

24 mask wearing.mp. (24)

25 Respiratory Tract Infections/pc, px, tm [Prevention & Control, Psychology, Transmission] (2796)

26 Influenza, Human/pc, px, tm [Prevention & Control, Psychology, Transmission] (12728)

27 Influenza A Virus, H1N1 Subtype/ or Influenza A Virus, H5N1 Subtype/ (12373)

28 Disease Outbreaks/ or pandemic flu.mp. (59045)

29 Influenza Vaccines/ (14488)

30 Vaccination/ (51248)

31 14 or 15 or 16 or 17 or 18 or 19 or 20 or 21 or 22 or 23 or 24 or 29 or 30 (125032)

32 25 or 26 or 27 or 28 (77763)

33 13 and 31 and 32 (137)

**CINAHL**

S33 S14 AND S18 AND S32 100

S32 S19 OR S20 OR S21 OR S22 OR S23 OR S24 OR S25 OR S26 OR S27 OR S28 OR S29 OR S30 OR S31 252,004

S31 (MH "Influenza Vaccine") OR "influenza vaccination" 6,469

S30 social isolation 4,961

S29 social distancing 47

S28 cough adj etiquette 1

S27 cough adj etiquette 0

S26 hand hygiene 1,574

S25 protective behavio* 690

S24 (MH "Masks") 1,107

S23 avoidance behavio* 499

S22 preventive behavio* 737

S21 non-pharmaceutical adj intervention 32

S20 non-pharmaceutical adj intervention 0

S19 (MH "Infection Control/UT/RF/PF/PC/MT/HI/EV") OR (MH "Handwashing") OR (MH "Patient Isolation") OR (MH "Quarantine") 10,248

S18 S15 OR S16 OR S17 18,556

S17 flu 4,432

S16 (MH "Influenza") OR "influenza" OR (MH "Influenza A Virus") OR (MH "Influenza, Avian") OR (MH "Influenza, Pandemic (H1N1) 2009") OR (MH "Influenza A H5N1") OR (MH "Influenza A Virus, H1N1 Subtype") OR (MH "Influenza, Human") OR (MH "Influenza, Seasonal") OR (MH "Influenza, Swine") OR (MH "Influenza A Virus, H5N1 Subtype") 17,030

S15 (MH "Respiratory Tract Infections/TM/TD/SS/RF/PF/PC/IM/HI") 997

S14 S1 OR S2 OR S3 OR S4 OR S5 OR S6 OR S7 OR S8 OR S9 OR S10 OR S11 OR S12 OR S13 108,929

S13 data saturation 309

S12 (MH "Purposive Sample") 15,623

S11 (MH "Content Analysis") 17,157

S10 (MH "Thematic Analysis") 25,620

S9 (MH "Discourse Analysis") 2,291

S8 (MH "Focus Groups") 21,346

S7 formative 1,512

S6 (MH "Participant Observation") 3,710

S5 (MH "Grounded Theory") 8,817

S4 (MH "Phenomenological Research") 8,354

S3 phenomenol* 11,699

S2 (MH "Ethnographic Research") OR "ethnography" 4,857

S1 (MH "Qualitative Studies") OR "qualitative" 66,876
